# One Word to Describe My Experience as a COVID-19 Survivor Six Months after Its Onset: Findings of a Qualitative Study

**DOI:** 10.3390/ijerph19094954

**Published:** 2022-04-19

**Authors:** Alvisa Palese, Maddalena Peghin, Valentina Bressan, Margherita Venturini, Valentina Gerussi, Giulia Bontempo, Elena Graziano, Erica Visintini, Carlo Tascini

**Affiliations:** 1 Department of Medical Science, University of Udine, Viale Ungheria, 20-33010 Udine, Italy; valentina.bressan@uniud.it (V.B.); margherita.venturini@uniud.it (M.V.); visintini.erica001@spes.uniud.it (E.V.); 2 Infectious Diseases Division, Department of Medicine, University of Udine and Azienda Sanitaria Universitaria Friuli Centrale (ASUFC), Piazzale Santa Maria della Misericordia, 15-33100 Udine, Italy; maddalena.peghin@asufc.sanita.fvg.it (M.P.); valentina.gerussi@asufc.sanita.fvg.it (V.G.); giulia.bontempo@asufc.sanita.fvg.it (G.B.); elenagraziano22@gmail.com (E.G.); carlo.tascini@asufc.sanita.fvg.it (C.T.)

**Keywords:** COVID-19, Coronavirus Disease 19, follow-up, lived experience, qualitative study, metaphors

## Abstract

The COVID-19 pandemic emotionally affected the lives of patients cared for in different settings. However, a comprehensive view of the whole experience as lived by survived patients, from the onset of the disease and over time, is substantially unknown to date. A descriptive qualitative design was implemented according to the Standards for Reporting Qualitative Research. Adult patients (=1067) cared for during the first wave (March/April 2020) capable of answering an interview and willing to participate were interviewed (=397) by phone with an interview guide including open- and closed-ended questions. In this context, they were asked to summarise with a metaphor their entire COVID-19 experience at six months. Then, the emotional orientation (positive, neutral, or negative) of the metaphors expressed was identified. The participants were mainly female (206; 51.9%), with an average age of 52.6 years (CI 95% 50.4–53.6), reporting a mild severity of COVID-19 disease at the onset (261; 65.7%) and the perception of being completely healed (294; 70%) at six months. The patients summarised their experiences mainly using negative-oriented (248; 62.5%) metaphors; only 54 (13.6%) reported positive-oriented metaphors and a quarter (95; 23.95) neutral-oriented metaphors. Nearly all positive-oriented metaphors were reported by patients with symptoms at the onset (53; 98.1%), a significantly higher proportion compared to those reporting negative- (219; 88.3%) and neutral–oriented (78; 82.1%) metaphors (*p* = 0.014). While no other clinical features of the disease were associated, among females, significantly more negative-oriented metaphors emerged. Moreover, neutral-oriented metaphors were reported by younger patients (49.5 years, CI 95% 64.11–52.92) as compared to those negative and positive that were reported by more mature patients (53.9; CI 95% 52.04–55.93 and 54.8; CI 95% 50.53–59.24, respectively) (*p* = 0.044). Nurses and healthcare services require data to predict the long-term needs of patients. Our findings suggest that, for many patients, the COVID-19 lived experience was negative over time.

## 1. Introduction

The recent Coronavirus Disease 19 (COVID-19) pandemic has been largely investigated regarding its clinical and emotional implications in different settings and at different stages of the disease trajectory among patients at home, during their in-hospital stay, and at their discharge [1,2,3]. However, few studies have investigated the meaning of the whole experience as lived by survived patients from the onset of the disease over time [4]. The recent appearance of the disease, as well as the lack of longitudinal studies, have been underlined as the main reasons for the lack of evidence regarding the consequences of COVID-19 [5] in the long term [6] that might negatively affect health-related quality-of-life (HRQoL) at the individual and at the family levels [7]. To advance the knowledge in the field, studies investigating a variety of conditions and populations with different designs have been strongly encouraged [5]. The main intent of this study was to contribute to this advancement, describing the meaning of the whole experience as lived by survived patients from the onset of COVID-19 disease up to six months.

### Background

Hospitalised COVID-19 patients have been reported to experience fear and stigma, with the major source of stress lying in the respiratory highly contagious viral nature of the disease, the isolation measures applied, and in concerns regarding the health of family relatives [8]. Moreover, they have been reported to experience extreme uncertainties during the initial stage of the diagnosis, complex negative feelings during the treatment stage, and then growth during the recovery [8,9].

Studies investigating the immediate post-discharge life have documented patients still in fear of being reinfected or of remaining infectious; however, they have been reported to be ready to adopt new behaviours, including hygiene practices, and changes in lifestyle (e.g., [3]). One month after hospital discharge, patients with severe COVID-19 disease have been reported to experience sequelae in their respiratory status and physical and mental health [10]. Moreover, 90 days after an intensive care unit discharge, their quality of life has been documented to be worsened both physically and psychologically [11]. More recently, follow-up studies have explored the health status as the persistence of symptoms (e.g., [12]) and their emotional implications [1], aiming at identifying the individual needs and the long-term consequences of the disease. Specifically, long COVID-19 patients have been reported to encounter difficulties in managing their symptoms and in accessing care; uncertainty, helplessness, and fear have also been documented [13]. As briefly reported, the studies available have involved different groups of patients in different stages of their disease [8,9,13,14]. However, their view of the whole experience as lived by survived patients, from the onset of the disease and over time, in different settings is substantially unknown to date [4].

The patients’ experiences (Patient Reported Experience Measures, PREMs [15]) have been reported among the indicators capable of exploring health outcomes and HRQoL determinants (e.g., [16]). Patient experiences might be collected around an episode of care—as, for example, during hospital discharge—and as a whole, from the disease onset. Collecting narratives openly over time, allowing patients to share the meaning of the lived experience from tragedy to the beneficial implications, might give insights into the different ways that humans live and adapt themselves to challenges [17]. Moreover, opening a dialogue with patients who have survived a global tragedy, such as the COVID-19 pandemic, by giving them a ‘voice’ might provide support in designing new services and in improving the quality of those offered, as well as in prioritising the services around patients’ needs. In addition, detecting the lived experiences of patients might also demonstrate the emotions that nurses and other front-line healthcare professionals need to be aware of and their reciprocal influences: in the early stage of the disease, healthcare professionals have been reported to live negative and positive experiences [18]. More recently, both positive and negative emotions have also been reported among front-line nurses, with mainly negative emotions in the first stage and positive emotions appearing gradually [19].

Ultimately, collecting their experiences might promote patient and the public’s participation in the development of future clinical and public health services. All these potential benefits of reporting patients’ experiences are of tremendous importance in the case of individuals who have survived COVID-19, given the need to transform both individual and collective experiences into an effective global learning [1,20,21]. In this context, Italy has been identified as the second country in the world [22] to have been dramatically affected by the pandemic [23]; thus, studies investigating the long-term implications among survived patients might also help other countries to anticipate some issues [2]. Therefore, the intention of this study was to describe the experiences of individuals who survived COVID-19, ranging from asymptomatic to severely ill, at six months after the disease onset.

## 2. Materials and Methods

### 2.1. Study Design

An explanatory sequential study design was performed in two phases: an initial quantitative phase was conducted, followed by a qualitative phase. The first longitudinal study started on March/April 2020; then, a descriptive qualitative design was performed in October/November 2020, according to its (a) capacity to gain insights from key informants on a not fully understood phenomenon [24]—as COVID-19 is—and (b) to develop knowledge regarding the whole experience of patients. Specifically, an inductive approach was employed, allowing data shared by survived patients to speak for themselves [15]. The methods and findings of the qualitative phase are reported here according to the Standards for Reporting Qualitative Research [25] (see Supplementary Table S1).

### 2.2. Setting and Participants

**Figure 1:**
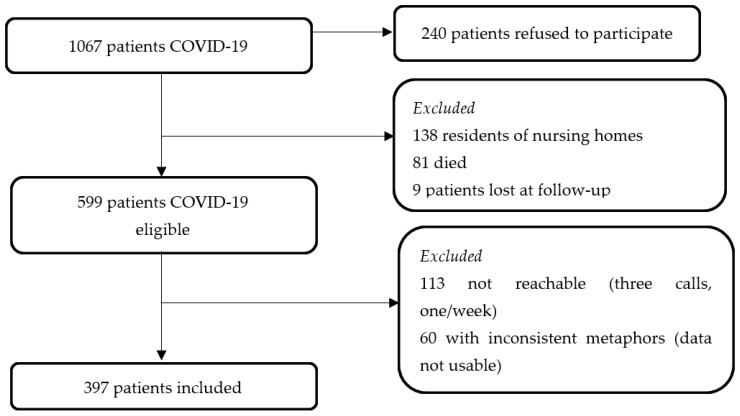
Flow chart showing patients diagnosed with COVID-19 interviewed six months after the onset. Legend. COVID-19, Coronavirus Disease 19.

The study was conducted by the Infectious Disease Unit of a large academic hospital (>1000 beds) in the Friuli Venezia Giulia Region (Italy) from March/April to October/November 2020. During the first wave of the COVID-19 outbreak, starting from 1 March 2020—the day when the first case was identified—to 30 May 2020, a total of 1067 patients were cared for and diagnosed with COVID-19, according to the positive nucleic acid amplification test (NAAT) for severe acute respiratory syndrome coronavirus 2 (SARS-CoV-2) in respiratory tract specimens. All of them composed the target population. A total of 240 patients refused to be involved in the study, while 138 residents of nursing homes were not involved mainly due to their cognitive decline; moreover, nine patients were lost in the follow-up, and 81 died. Therefore, 599 patients were eligible. Among them, those included in the qualitative phase were (a) adults (>18 years), (b) reachable by telephone, (c) at six months after their disease onset, and (d) willing to participate in a telephone interview. A total of 202 patients were excluded and 397 were invited to participate, as reported in Figure 1.

### 2.3. Data Collection Instrument and Method

Data collection was performed at two time points for each eligible patient: at the COVID-19 onset and after six months, with an interview guide developed from the available literature [26,27,28,29,30]. Specifically, at the disease onset, (a) sociodemographic (e.g., age and gender) and (b) clinical data (e.g., severity of COVID-19, hospitalisation (yes/no), length of in hospital stay, comorbidities, and symptoms (number and nature)) were collected and populated in a database. The severity of the COVID-19 disease was expressed as: asymptomatic; mild disease (without pneumonia); moderate disease (pneumonia); severe disease (severe pneumonia); or critical disease, including acute respiratory distress syndrome (ARDS), sepsis, and/or septic shock [27]. Moreover, among others, dyspnoea was identified as an important symptom not only for its relevance in the context of COVID-19 but also according to its implications emotionally and socially and the activities of daily living (ADL), thus affecting the whole experience [28].

Then, at six months, an interview guide composed of open-ended and closed questions was developed and aimed at collecting data regarding: (a) the source of the contagion (e.g., family members); (b) the perception of being healed at six months, and, if any, the persisting symptoms; and (c) a metaphor or a word summarising the entire COVID-19 experience at six months. For each patient, time was left to reflect and to summarise his/her personal experience. Specifically, the last question was open-ended, and each patient was left free to share a symbolic summary of her/his experience by using a metaphor. The use of metaphors, or words capable of condensing a meaning, has been suggested to shed light on aspects of a phenomenon not previously documented [29], as well as to portray complex realities [30], such as living with COVID-19. Moreover, metaphors have been suggested as being useful in sharing the meaning of a lived experience [31]. Asking patients to summarise their lived experiences by using a metaphor triggers a sort of identification and categorisation process [32] through a linguistic device [33]. Given their function as figures of speech, metaphors constitute the root of human knowledge [32] and can be considered not simply as the outcome of an experience but as ‘a powerful metaphor [that] initiates and guides social processes’ [34].

We asked patients to express metaphors/words summarising the entire COVID-19 experience (hereinafter, metaphors) in an attempt to (a) reduce the data: collecting metaphoric labels to epitomise the meaning of a given experience has been documented as effective in summarising a range of meanings [30]; (b) merge their main orientation (positive, negative, or neutral) as the prevailing emotion embodied in the metaphor expressed [35], thus enhancing their fundamental role in representing the lived experience of patients; and (c) count their frequency to compare the meaning [35] across different demographic and clinical profiles of patients.

The interview guide was firstly piloted for feasibility, clarity, and understandability among 10 patients not involved in the final analysis. No changes were suggested.

### 2.4. Data Collection Rigour

Firstly, patients were approached during in-hospital follow-ups where the aims of the study were presented and informed consent to be interviewed was obtained. Participants were then contacted by three nurses who interviewed them via telephone around six months after the onset: in a preliminary fashion, they informed again each participant regarding the aims of the study and the procedures. Then, the best moment for the interview with the patient was agreed upon, and the interview was also reallocated to different days/hours when requested. Each participant was left free to answer with his/her own words. The metaphor was recorded in the patient database and then repeated by the researcher who interviewed the patient to ensure its accuracy. Moreover, in case of persisting symptoms or issues, researchers contacted the Infectious Disease Units to refer the patients in need of a visit.

The researchers involved in the data collection (EV, VB, and MV) were all female, well-educated (at a PhD level and at a Master’s level of Nursing Science), with experience in (a) research methodology, (b) clinical care, and (c) teaching. Those involved in the research protocol development (AP, MP, GB, VG, EG, and CT) and in the data analysis were female and male researchers that were experts in research and infectious diseases where they were working at the time of the study.

The researchers had no previous relationship with the patients, and when presumed (e.g., similar age/town of provenance), a different researcher in both age and provenance was involved in the data collection. Moreover, given that the data analysis was performed by all members of the project team, the personal identification of the patients was blinded to prevent any form of identification by those researchers working in the Infectious Disease Unit and, thus, involved in the care of patients during the first wave. Given that no previous experience with these patients was accounted for, the researchers reported no potential assumptions and/or presuppositions before the data collection and analysis.

In order to prevent selection bias [36], the main demographic and clinical characteristics were compared between those involved and those who did not take part in the interview; an information bias [36] was prevented by involving patients after six months from their disease onset during a period when the experience was still recent and when rates of post-traumatic stress disorders have been documented as being diminished; moreover, by performing the interviews after six months, recall bias was prevented homogeneously across the participants [36].

### 2.5. Data Analysis

A descriptive statistical analysis was performed for the demographic and clinical variables (frequencies, percentages, averages, and confidence intervals (CI) at 95%). Then, all the metaphors recorded were transcribed into a file and checked for completeness. Next, these were firstly counted, and then:

(a) Selected: For those patients who expressed more than one metaphor, one was identified according to its intensity and capacity to condense the meaning of the whole experience.
(b) Summarised: All metaphors were summarised in a single word expressing the ‘metaphor vehicle’ as the prototypical example of a given category [31]. A content analysis of each metaphor [37] was performed and then compared with each other. Their metaphorical qualities were checked; while some maintained their structure (e.g., ‘Nightmare’), others expressed mainly a process (e.g., ‘Rethinking’) or a feeling (e.g., ‘Fear’ or ‘Concern’). However, given that all the expressions that emerged reflected how patients categorise and make sense of their lived experience(s), all were retained as being able to express the quality required by the metaphors [31].
(c) Categorised [35]: All were categorised into their emotional orientation (positive, neutral, or negative) according to the context of the expression reported by each participant.
(d) Analysed: A corroborative counting technique was performed [38] using a free web program [39] considering the metaphor orientation (positive, negative, and neutral). Specifically, we used a free Word Cloud analysis to detect the frequency of the metaphors according to their orientation. This method identifies the most common words found in a data set and displays them in a cloud where the frequency of the methods (in our case) is indicated by the size of the font used to display the word [40]. Then, a descriptive analysis of the metaphors according to their orientation was performed [41]. Furthermore, a comparison within the same metaphor orientation was conducted across the different main characteristics of the patients using the chi-square test (χ^2^) for dichotomous variables. The statistical differences were set at *p* < 0.05. Data were analysed with SPSS version 26. The entire process was performed for all the metaphors expressed in an independent fashion by the three researchers; then, they agreed upon the findings to ensure an investigator triangulation of the data analysis [42].

## 3. Results

### 3.1. Participants

As reported in Table 1, among the 397 patients interviewed, the majority were female (206; 51.9%) and Italian (366; 92.2%) and educated through high school (160; 40.3%), with an average age of 52.6 years (CI 95% 50.4–53.6). Most of them (261; 65.7%) reported a mild severity of COVID-19 disease at the onset. The interviewed patients were younger compared to those not interviewed (52.6 vs. 59.6 years), and a few (22; 5.5% vs. 42; 7.2%) reported severe disease; the interviewed and non-interviewed patients were instead homogeneous for gender distribution and hospitalisation (see Supplementary Table S2). The duration of hospitalisation was, on average, 10.1 days (CI 95% 8.0–12.3).

At the onset of COVID-19, most patients reported comorbidities (207; 52.1%); according to their narratives, they were infected mainly by family members (229; 57.7%), and the disease appeared with symptoms (350; 88.2%); on average, >4 (CI 95% 4.51–5.0). Dyspnoea was reported by 32.6% of cases.

After six months, the majority reported to perceive themselves completely recovered (294; 70%), and one-third reported still having some symptoms (128; 32.3%), as shown in Table 1.

### 3.2. Metaphors

The patients summarised their experiences by using mainly one metaphor (254; 64%), but more than one-quarter of them used two (106; 26.7%). A limited number of them used three (32; 8.1%), four (4; 1.0%), or five (1; 0.2%) metaphors.

Most of the metaphors were negative-oriented (248; 62.5%), with ‘Fear’, ‘Bad’, and ‘Nightmare’ more often reported. Only 54 (13.6%) patients reported positive-oriented metaphors, with ‘Rediscovery’ and ‘A time for thinking’ the most cited. Moreover, around one-quarter of patients (95; 23.95) used neutral-oriented metaphors, neither positive nor negative, thus describing their experience as ‘Surreal’ or ‘Unexpected’ (Table 2 and Supplementary Table S3).

A free Word Cloud analysis was used to detect the frequency of the metaphors according to their orientation. This method identifies the most common words found in a data set and displays them in a cloud where the frequency of the methods (in our case) is indicated by the size of the font used to display the word [39,40]. Survived females reported negative-oriented metaphors more often (148; 59.7%) compared to males, who reported neutral (60; 63.2%) or positive-oriented ones more often (31; 57.4%) (*p* < 0.001). Moreover, neutral-oriented metaphors were reported by younger patients (49.5 years, CI 95% 64.11–52.92) as compared to negative and positive-oriented metaphors, which were reported by more mature patients (53.9; CI 95% 52.04–55.93 and 54.8; CI 95% 50.53–59.24, respectively) (*p* = 0.044). Nearly all positive-oriented metaphors were reported by patients with symptoms at the onset of COVID-19 (53; 98.1%), a significantly higher proportion compared to those reporting negative- (219; 88.3%) and neutral–oriented (78; 82.1%) metaphors (*p* = 0.014). As reported in Table 3, no other statistical significances emerged according to the profiles of the patients included.

## 4. Discussion

### 4.1. Patients

We interviewed adult Italian individuals who survived COVID-19 six months after the disease onset, which mainly appeared with symptoms and was then diagnosed as mild disease; for most participants, hospitalisation was not required, and they reported to have been infected mainly at home from family members. Moreover, the patients perceived themselves as mainly healed after six months, while one-third reported that they were still suffering from the symptoms. Therefore, the COVID-19 experience that emerged reflected survived patients with comprehensively positive outcomes. To the best of our knowledge, the qualitative studies available have investigated the early experience, during hospitalisation [8,9], isolation [3,14], or as perceived by nurses [43]. Moreover, follow-up studies have been performed with quantitative methods at four weeks [44], three months [45], or at six months, mainly regarding symptoms [6]. Furthermore, to the best of our knowledge, no studies have been performed to date asking patients to express a metaphor summarising their whole COVID-19 experience after six months, a methodology that has been used a few times in healthcare settings (e.g., [46]). Metaphors have been suggested to help in giving a name to something that we know intuitively and to describe an experience that is difficult to label [45,47], such as being an individual who survived COVID-19 might be.

### 4.2. Metaphors

Patients were able to summarise their experiences with, on average, one or two words, but some used from three or up to five, suggesting the richness and complexity of their experiences. At six months, the used metaphors were mainly negative, which seems to express the persistence of a negative implication of the pandemic experience already documented among the general population in several countries (e.g., China, Italy, Iran, and Spain), with high rates of anxiety, depression, post-traumatic stress disorders, psychological distress, and stress [48]. On the other hand, survived patients might have reported mainly negative-oriented metaphors according to their increased vulnerability as a result of stigmatisation and/or socioeconomic difficulties and the limited support received [49]. In this context, COVID-19 has been underlined as a traumatic stressor event triggering post-traumatic stress disease-like responses and exacerbating some mental health issues (e.g., anxiety); moreover, researchers have also suggested the existence of COVID-19 unique disorders named ‘COVID stress syndrome’ [50], suggesting the need to continually follow-up on patients not only for their clinical problems but also regarding their emotional implications from the stress experienced.

A few survived patients reported a positive-oriented experience: the COVID-19 pandemic has also been seen to trigger positive transformations, namely post-traumatic growth [51], such as positive changes and achievements due to the ability to cope with adversity. Among these survived patients, it seems that the crisis has provided a special occasion for meaning and for turning the life-threatening event into an opportunity; in other words, in moving from a loss towards a gain, achieving a new adjustment [52]. Interestingly, nearly all participants using positive-oriented metaphors reported symptoms at the onset of COVID-19 and mild disease; thus, they might perceive themselves as having been at increased risk of losing their life. Similar findings have been documented recently by using qualitative methods among 40 Chinese individuals who survived COVID-19. In summarising the experience, the authors reported a re-evaluation of the patients’ life priorities, an attempt to establish improved relationships, closer relationships with family and friends, and a greater willingness to help others. Moreover, patients were reported to perceive changes regarding themselves, which included personal growth and increased awareness of the importance of their health [8]. Similar to previous pandemics, the investigations concerning COVID-19 have mainly focused on the negative emotional implications [8]; however, patients experiencing traumatic events might also grow and report potential positive experiences that should be discovered when using an underlying mechanism in order to identify strategies aimed at promoting such growth. Continuing to follow-up the survivors at 12 months might help in understanding whether those who reported negative-oriented metaphors after six months of the disease change their perspectives. Negative emotions have been reported as being dominant during the early stages [8,9].

One-quarter of participants summarised their survived experience by using neutral-oriented metaphors. The ‘lived experience of the illness’ has been included as an important dimension in chronic patient experiences, requiring continuous psychosocial adjustment to the disease, with positive or negative adaptations [53]. Survived patients might perceive themselves as completely healed after an acute disease, lived as a ‘normal’ episode of life. Moreover, survived patients might have maintained their own internal balance despite the experiences of traumatic events or stressful conditions [54] not only lived individually but also within the family and collectively, such as a pandemic might trigger. No clinical factors, such as the severity of the disease at the onset or being hospitalised or not or having an increased number of symptoms, among them dyspnoea, have been associated with a metaphor orientation. Similarly, the persistence or not of the symptoms or feeling healed at six months have also been associated with the metaphor orientation. This seems to suggest that the disease is lived according to the personal resources of the individuals, to the support they receive, and to their capacity to give meaning to the crisis in their life and not according to the clinical issues. Personal resources might also be affected by the brain sequels of COVID-19 that have been documented as affecting a large proportion of patients—up to 70%—and in the long term after the respiratory symptoms are resolved [55].

Substantially, only two individual factors have been associated with negative-, positive-, or neutral-oriented metaphors. Female gender was associated with a higher occurrence of negative-oriented metaphors, in line with previous studies [23,45], suggesting that they are in greater need of help. Moreover, the ages were, on average, higher among those who reported positive- and negative-oriented metaphors compared to those who reported neutral-oriented ones. Regarding the positive orientation, the evidence highlighted that older individual are more skilled in coping with stressful situations than younger people, according to their life experiences [56] and their better emotional regulation [57]; on the other hand, regarding the negative-oriented metaphors, our findings seem to suggest that they are more vulnerable. However, the practical meaning of the statistical significance that emerged in our study is limited, given that the average ages were similar across the groups; therefore, future investigation in this area is required.

### 4.3. Study Limitations

The study was designed as descriptive-focused to detect the main metaphor orientation and develop potential practical implications; therefore, it was not intended to contribute to the theory development in this field. A selection bias [36] might have affected the findings, given that only 397 out of 1097 patients were interviewed, and 240 refused to participate. All the strategies were adopted to prevent biases in the data collection by contacting those patients not reachable via phone more times and by comparing the main profiles of those interviewed with those who did not participate or who had died. We invited patients to express their experiences with one word or with a few words, according to the underpinning theories [29,30,31,32,34]: their capacity to summarise the meaning of the experience might have been affected by education and by their well-being perceived during the post-COVID-19 time [58] as influenced also by the care quality received [59,60]. Moreover, the metaphors were summarised in a single word expressing the ‘metaphor vehicle’ as the prototypical exemplar of a given category [31]; some of them maintained their metaphoric qualities (e.g., ‘Nightmare’), while others expressed processes (e.g., ‘Fortifying’) or feelings (e.g., ‘Unexpected’). Furthermore, the translation of words from Italian into English may have influenced their meaning; to prevent this, the translation was made by expert nurses involved as the interviewer, thus knowing the original meaning. However, the interest was in the orientation of such metaphors and not their specific meaning that might be interpreted not only linguistically but also within the sociocultural context of each individual. Additionally, there was not a control group including citizens who did not suffer from COVID-19 in order to detect, among them, the whole pandemic experience; similar experiences as those lived by relatives according to their proximity to a patient who suffered from the disease might also be interesting to investigate further.

## 5. Conclusions

We involved a large group of an unselected population—from asymptomatic to those with severe disease—affected by COVID-19 during the first wave in Italy in an attempt to capture the whole experience as perceived by them at six months after the onset.

The lived experiences in the first six months were mainly summarised with negative-oriented metaphors, suggesting the persistence of negative implications of the pandemic experience on their whole lives. One-quarter of the participants used neutral-oriented metaphors, while a few remarked on a positive-oriented experience. No clinical factors influenced these perceptions, suggesting that the disease is lived according to personal resources, to the capacity to give a meaning to a crisis in life, and to the support received. However, females reported mainly a negative-oriented experience, suggesting a higher vulnerability and, thus, the need for increased follow-up and support. On the other hand, the ages were, on average, higher among those who reported positive- and negative-oriented metaphors compared to those who reported neutral-oriented ones, a finding that should be further investigated.

Nurses and healthcare services require data to predict the long-term needs of patients; our findings suggest that, for several patients, their well-being might be negatively affected by COVID-19 over time. Therefore, supportive programmes helping them to overcome their negative experiences, as well as easy access to services when required, is suggested.

Further, discovering the meaning of the experience at one year and over time with the intent to detect trends, if any, might also add support to identifying any long-term needs. Additionally, discovering the underlying strategies enabling a possible transformation from negative- to positive-oriented metaphors, thus reflecting growth, seems to be crucial in tailoring the support of patients at risk of living this experience negatively in the long term.

## Figures and Tables

**Table 1:** Characteristics of the interviewed patients.

At the COVID-19 Onset	*N* = 397 (%)
Gender	
Female	206 (51.9)
Male	191 (48.1)
Age (years), mean (CI 95%)	52.6 (50.4–53.6)
Nationality	
Italian	366 (92.2)
Non-Italian	31 (7.8)
Education	
None	1 (0.3)
Primary School	22 (5.5)
Middle School	65 (16.4)
High School	160 (40.3)
Bachelor’s Degree	83 (20.9)
Ph.D.	3 (0.8)
Missing	63 (15.9)
COVID-19 severity, WHO scale [27]	
Asymptomatic	51 (12.8)
Mild disease (without pneumonia)	261 (65.7)
Moderate disease (pneumonia)	60 (15.1)
Severe disease (severe pneumonia)	13 (3.3)
Critical disease, including acute respiratory distress syndrome (ARDS), sepsis and/or septic shock	9 (2.2)
Missing	3 (0.8)
Hospitalised for COVID-19	
Yes	101 (25.4)
Department(s)/units ^§^	
Infectious Disease	83 (82.2)
COVID-19	17 (16.8)
Intensive Care	14 (13.9)
Pneumology	11 (10.9)
Hospitalisation (days), mean (CI 95%)	10.1 (8.0–12.3)
Previous comorbidities	
Yes	207 (52.1)
Infected by whom	
I don’t know	128 (32.2)
Family members	229 (57.7)
Colleagues	32 (8.1)
Family members and colleagues	8 (2.0)
COVID-19 symptoms at the COVID-19 onset	
Yes	350 (88.2)
No	47 (11.8)
Symptoms, number, mean (CI 95%)	4.75 (4.51–5.0)
Among symptoms, dyspnoea	114 (32.6)
At six months after the COVID-19 onset	
Feeling completely healed	
Yes	294 (70.0)
No	88 (22.2)
Uncertain	15 (3.8)
Persisting COVID-19 symptoms	
Yes	128 (32.2)
No	250 (63.0)
Uncertain	19 (4.8)

Legend: CI, confidence interval; COVID-19, Coronavirus Disease 19; N, number; Ph.D., Doctor of Philosophy; SD, standard deviation; WHO, World Health Organization: asymptomatic; mild disease (without pneumonia); moderate disease (pneumonia); severe disease (severe pneumonia); critical disease, including acute respiratory distress syndrome (ARDS), sepsis, and/or septic shock [27]. ^§^ Some patients were hospitalised in more than one department.

**Table 2:** ‘My lived experience as a COVID-19 survived patient’: metaphors and their orientation (=397).

Negative-Oriented 248 (62.5%)	Neutral-Oriented 95 (23.9%)	Positive-Oriented 54 (13.6%)
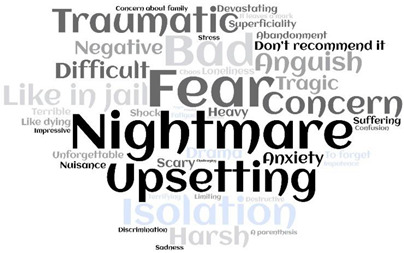	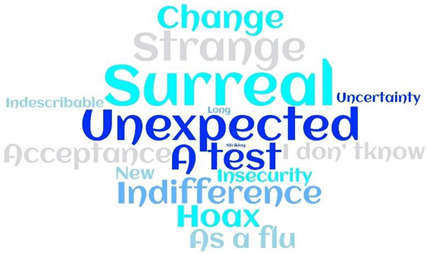	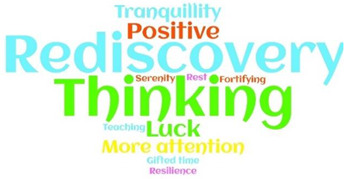

Legend: COVID-19, Coronavirus Disease 19.

**Table 3:** Metaphors’ orientations, according to the patients’ characteristics and over time.

At the COVID-19 Onset	Negative-Oriented	Neutral-Oriented	Positive-Oriented	*p*-Value
	*N* = 248 (%)	*N* = 95 (%)	*N* = 54 (%)	
Gender				<0.001
Female	148 (59.7)	35 (36.8)	23 (42.6)	
Male	100 (40.3)	60 (63.2)	31 (57.4)	
Age (years), mean (CI 95%)	53.9 (52.04–55.93)	49.5 (46.11–52.92)	54.8 (50.53–59.24)	0.044
Nationality				
Italian	227 (91.5)	88 (92.6)	51 (94.4)	0.754
Not Italian	21 (8.5)	7 (7.4)	3 (5.6)	
Education	208/248	79/95	47/54	
None	1 (0.5)	0 (-)	0 (-)	0.94
Primary School	13 (6.3)	6 (7.6)	3 (6.4)	
Middle School	38 (18.3)	18 (22.8)	9 (19.1)	
High School	98 (47.1)	39 (49.4)	23 (48.9)	
Bachelor’s Degree	55 (26.4)	16 (20.3)	12 (25.5)	
Ph.D.	3 (1.4)	0 (-)	0 (-)	
Severity of COVID-19 disease, WHO scale [27]				
Asymptomatic	30 (12.1)	14 (14.7)	7 (13.0)	0.537
Mild disease (without pneumonia)	162 (65.3)	65 (68.4)	34 (63.0)	
Moderate disease (pneumonia)	39 (15.7)	15 (15.8)	6 (11.1)	
Severe disease (severe pneumonia)	8 (3.2)	1 (1.1)	4 (7.4)	
Critical disease, including acute respiratory distress syndrome (ARDS), sepsis and/or septic shock	7 (2.9)	0 (-)	2 (3.7)	
Missing	2 (0.8)	0 (-)	1 (1.8)	
Hospitalised for COVID-19	65 (26.2)	22 (23.2)	12 (24.1)	0.827
In Intensive Care Units	10 (4.0)	1 (1.1)	3 (5.6)	0.28
Hospitalisation (days), mean (CI 95%)	9.9 (7.67–12.15)	7.19 (2.08–12.30)	12.14 (2.59–21.70)	0.396
Infected by whom				0.22
I don’t know	76 (30.6)	34 (35.8)	18 (33.3)	
Family members	152 (61.3)	50 (52.6)	27 (50)	
Colleagues	16 (6.5)	10 (10.5)	6 (11.1)	
Family members and Colleagues	4 (1.6)	1 (1.1)	3 (5.6)	
COVID-19 symptoms at the onset				
Yes	219 (88.3)	78 (82.1)	53 (98.1)	0.014
Symptoms, number, mean (CI 95%)	4.85 (4.54–5.15)	4.51(4.03–5.00)	4.74 (4.01–5.46)	0.54
Among symptoms, dyspnoea	73 (29.4)	26 (27.4)	15 (27.8)	0.918
Comorbidities	137 (55.5)	46 (48.4)	24 (44.5)	0.234
At six months after COVID-19 onset				
Feeling healed/recovered				
Yes	183 (76.3)	69 (78.4)	42 (77.8)	0.908
No	57 (23.7)	19 (21.6)	12 (22.2)	
Persisting COVID-19 symptoms				
Yes	156 (65.0)	55 (64.0)	39 (75.0)	0.342
No	84 (35.0)	31 (36.0)	13 (25.0)	

Legend: CI, confidence interval; COVID-19, Coronavirus Disease 19; N, number; Ph.D., Doctor of Philosophy; SD, standard deviation; WHO, World Health Organization: asymptomatic; mild disease (without pneumonia); moderate disease (pneumonia); severe disease (severe pneumonia); critical disease, including acute respiratory distress syndrome (ARDS), sepsis, and/or septic shock [27].

## Data Availability

Data available on request due to restrictions, e.g., privacy or ethical.

## Supplementary Materials

The following supporting information can be downloaded at https://www.mdpi.com/article/10.3390/ijerph19094954/s1: Table S1: Standards for Reporting Qualitative Research (SRQR). Table S2: Patients interviewed, not interviewed, and died: main profiles. Table S3. ‘*My lived experience as a COVID-19 survived patient*’: metaphors according and their orientation (=397).
