# Supplementary material for: One Word to Describe My Experience as a COVID-19 Survivor Six Months after Its Onset: Findings of a Qualitative Study
Source: Int J Environ Res Public Health. 2022 Apr 19;19(9):4954. doi: 10.3390/ijerph19094954 (PMC9106002; doi:10.3390/ijerph19094954)
Supplement: Supplementary file 1 [file ijerph-19-04954-s001.zip › ijerph-1614968-supplementary.pdf]

Supplementary Table S1. Standards for Reporting Qualitative Research (SRQR) [25]

Title and abstract	Section
**Title -** Concise description of the nature and topic of the study Identifying the study as qualitative or indicating the approach (e.g., ethnography, grounded theory) or data collection methods (e.g., interview, focus group) is recommended	Title
**Abstract -** Summary of key elements of the study using the abstract format of the intended publication; typically includes background, purpose, methods, results, and conclusions	Abstract
**Introduction**	
**Problem formulation -** Description and significance of the problem/phenomenon studied; review of relevant theory and empirical work; problem statement	Introduction Background
Purpose or research question - Purpose of the study and specific objectives or questions	Introduction Background
**Methods**	
Qualitative approach and research paradigm - Qualitative approach (e.g., ethnography, grounded theory, case study, phenomenology, narrative research) and guiding theory if appropriate; identifying the research paradigm (e.g., postpositivist, constructivist/ interpretivist) is also recommended; rationale**	Study design
Researcher characteristics and reflexivity - Researchers' characteristics that may influence the research, including personal attributes, qualifications/experience, relationship with participants, assumptions, and/or presuppositions; potential or actual interaction between researchers' characteristics and the research questions, approach, methods, results, and/or transferability	Data collection instrument and method<br>Data collection rigour
Context - Setting/site and salient contextual factors; rationale**	Participants and setting
Sampling strategy - How and why research participants, documents, or events were selected; criteria for deciding when no further sampling was necessary (e.g., sampling saturation); rationale**	Participants and setting<br>Figure 1
Ethical issues pertaining to human subjects - Documentation of approval by an appropriate ethics review board and participant consent, or explanation for lack thereof; other confidentiality and data security issues	Institutional Review Board Statement<br>Informed Consent Statement
Data collection methods - Types of data collected; details of data collection procedures including (as appropriate) start and stop dates of data collection and analysis, iterative process, triangulation of sources/methods, and modification of procedures in response to evolving study findings; rationale**	Data collection instrument and method
Data collection instruments and technologies - Description of instruments (e.g., interview guides, questionnaires) and devices (e.g., audio recorders) used for data collection; if/how the instrument(s) changed over the course of the study	Data collection instrument and method
Units of study - Number and relevant characteristics of participants, documents, or events included in the study; level of participation (could be reported in results)	Results, Table 1, Supplementary Table 2

Data processing - Methods for processing data prior to and during analysis, including transcription, data entry, data management and security, verification of data integrity, data coding, and anonymization/de-identification of excerpts	Data analysis
Data analysis - Process by which inferences, themes, etc., were identified and developed, including the researchers involved in data analysis; usually references a specific paradigm or approach; rationale**	Data analysis
Techniques to enhance trustworthiness - Techniques to enhance trustworthiness and credibility of data analysis (e.g., member checking, audit trail, triangulation); rationale**	Data collection rigour
**Results/findings**	
Synthesis and interpretation - Main findings (e.g., interpretations, inferences, and themes); might include development of a theory or model, or integration with prior research or theory	Results
Links to empirical data - Evidence (e.g., quotes, field notes, text excerpts, photographs) to substantiate analytic findings	
**Discussion**	
Integration with prior work, implications, transferability, and contribution(s) to the field - Short summary of main findings; explanation of how findings and conclusions connect to, support, elaborate on, or challenge conclusions of earlier scholarship; discussion of scope of application/generalizability; identification of unique contribution(s) to scholarship in a discipline or field	Discussion
**Limitations** - Trustworthiness and limitations of findings	Limitations<br>Data collection rigour
**Other**	
Conflicts of interest - Potential sources of influence or perceived influence on study conduct and conclusions; how these were managed	Data collection rigour
Funding - Sources of funding and other support; role of funders in data collection, interpretation, and reporting	None

*The authors created the SRQR by searching the literature to identify guidelines, reporting standards, and critical appraisal criteria for qualitative research; reviewing the reference lists of retrieved sources; and contacting experts to gain feedback. The SRQR aims to improve the transparency of all aspects of qualitative research by providing clear standards for reporting qualitative research.

**The rationale should briefly discuss the justification for choosing that theory, approach, method, or technique rather than other options available, the assumptions and limitations implicit in those choices, and how those choices influence study conclusions and transferability. As appropriate, the rationale for several items might be discussed together.

Supplementary Table S2 Patients interviewed, not interviewed, and died: main profiles

**Patients COVID-19 (N=1097)**	**Interviewed N= 397 (%)**	**Not interviewed N= 588 (%)**	**Died N= 81 (%)**	**p-value** (interviewed vs. not interviewed)
Gender				
Female	206 (51.9)	337 (57.3)	52 (64.2)	0.090
Male	191 (48.1)	251 (42.7)	29 (35.8)	
Age (years), mean (CI95%)	52.6 (50.4-53.6)	59.6 (57.6-61.74)	83.2 (81.0-85.5)	<0.001
Severity of the disease according to WHO scale [27] §				<0.001
Asymptomatic	51 (12.8)	77 (13.1)	0 (-)	
Mild disease (without pneumonia)	261 (65.7)	222 (37.8)	3 (3.7)	
Moderate disease (pneumonia)	60 (15.1)	65 (11.1)	7 (8.6)	
Severe disease (severe pneumonia)	13 (3.3)	27 (4.6)	19 (23.5)	
Critical disease (acute respiratory distress	8 (2.0)	14 (2.4)	15 (18.5)	
syndrome, sepsis and/or septic shock	1 (0.2)	1 (0.2)	1 (1.2)	
Missing	3 (0.8)	182 (31.0)	36 (44.4)	
Hospitalized for COVID-19	101 (25.4)	136 (23.1)	81 (100)	0.417

Abbreviations: CI, confidence interval; COVID-19, COronaVIrus Disease 19; SD, standard deviation; WHO, World Health Organisation.

§WHO scale, asymptomatic; mild disease (without pneumonia); moderate disease (pneumonia); severe disease (severe pneumonia); critical disease, including acute respiratory distress syndrome (ARDS), sepsis and/or septic shock [28].

Supplementary Table S3. '*My lived experience as a COVID-19 survived patient*': metaphors according and their orientation (=397).

Negative-oriented 248 (62.5%)	Neutral-oriented 95 (23.9%)	Positive-oriented 54 (13.6%)
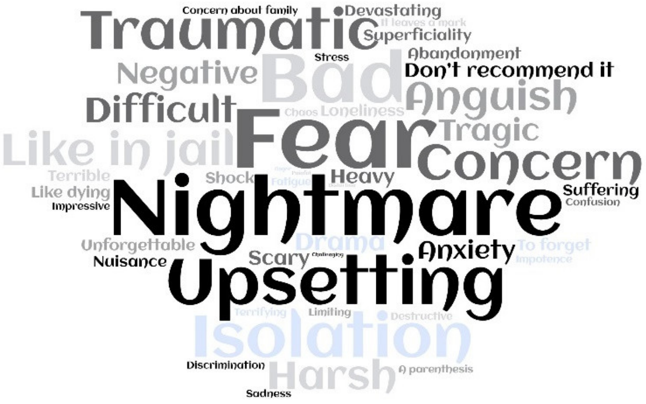	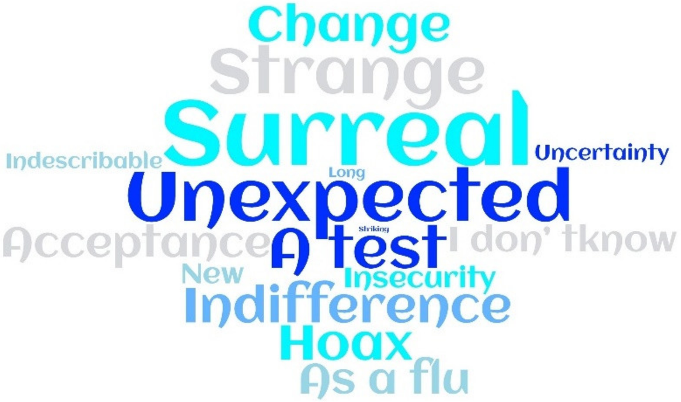	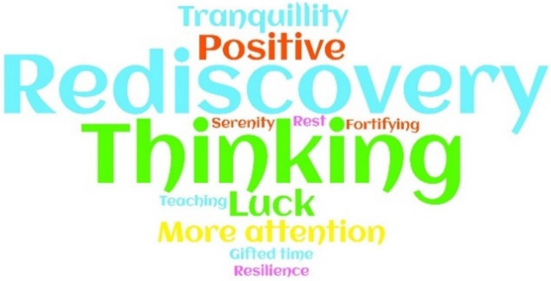

Metaphors	*n*	Metaphors	*n*	Metaphors	*n*
Fear	23	Surreal	13	Rediscovery (myself)	12
Bad…bad	17	Unexpected	10	A time for thinking	12
Nightmare	17	Strange	9	Positive	5
Upsetting	16	A test	8	Lucky	5
Isolation	15	Hoax	7	More attention (to myself)	4
Traumatic	11	Indifference	7	Tranquillity	4
Concern	10	Change	7	Resilience	2
Like in jail	9	Acceptance	6	Gifted time	2
Harsh	9	Like flu	6	Serenity	2
Anguish	9	I don't know	5	Teaching	2
Difficult	8	Insecurity	4	Fortifying	2
Tragic	8	New	4	Rest	2
Negative	6	Uncertainty	3		
Drama	6	Indescribable	3		
Anxiety	6	Long	2		
Don't recommend it	5	Striking	1		
Heavy	4				
Scary	4				

Loneliness	3
Abandonment	3
Devastating	3
Unforgettable	3
Terrible	3
Superficiality	3
Like dying	3
Nuisance	3
Shock	3
Suffering	3
To forget	3
Sadness	2
Limiting	2
Discrimination	2
It leaves a mark	2
Chaos	2
Impotence	2
Concern about family	2
Confusion	2
A parenthesis	2
Stress	2
Fatigue	2
Destructive	2
Impressive	2
Terrifying	2
Challenging	1
Distortion	1
Painful	1
Anger	1

Legend. COVID-19, Coronavirus Disease 2019.
